# Comparison of prepartum blood parameters in dairy cows with postpartum ketosis and new risk prediction candidates

**DOI:** 10.3389/fvets.2023.1161596

**Published:** 2023-05-11

**Authors:** Woojae Choi, Younghye Ro, Eunhui Choe, Leegon Hong, Dohee Kim, Seongdae Kim, Ilsu Yoon, Danil Kim

**Affiliations:** ^1^Department of Farm Animal Medicine, College of Veterinary Medicine, Seoul National University, Seoul, Republic of Korea; ^2^Farm Animal Clinical Training and Research Center, Institutes of Green-Bio Science and Technology, Seoul National University, Pyeongchang, Republic of Korea; ^3^Research Institute for Veterinary Science, Seoul National University, Seoul, Republic of Korea

**Keywords:** Periparturient period, ketosis, preventative blood parameters, lipid metabolism, osteocalcin, dairy cows

## Abstract

**Introduction:**

Ketosis is a predominant metabolic problem and a risk factor for several postpartum diseases. This retrospective study aimed to evaluate the complete blood count (CBC), plasma biochemistry, and osteocalcin and identify significant prepartum and early postpartum values expressed in ketotic cows.

**Methods:**

In 135 Holstein Friesian cows, 210 parturitions of 114 primiparous and 96 multiparous cows were examined. According to the plasma concentrations of β-hydroxybutyrate (BHB; ≥ 1.4 mmol/L) or non-esterified fatty acids (NEFA; ≥ 0.7 mmol/L) in the postpartum period, cows were divided into healthy cows (CON) and ketotic cows (KET). Analyses of CBC and biochemistry profiles were performed from −6 to 4 weeks of parturition every 2 weeks (prepartum; BW–5, BW–3, and BW–1, postpartum; BW1 and BW3), and osteocalcin ELISA tests were performed using blood samples from −2 to 2 weeks of parturition (BW–1 and BW1).

**Results:**

In primiparous KET (*n* = 114) before parturition, lower lymphocyte (Lym) in BW–5 and BW–3, lower red blood cell (RBC) in BW–5, higher mean corpuscular volume (MCV) in BW–1, and higher NEFA in BW–3 were significant compared with CON. Primiparous KET showed lower carboxylated osteocalcin (cOC) levels and a significant decrease after parturition. In multiparous KET (*n* = 96) before parturition, lower neutrophil (Neu) in BW–5, higher hemoglobin (HGB) in BW–5, higher MCV in BW–5 and BW–1, higher MCH in BW–5, lower total cholesterol (TC) in BW–5, higher triglyceride (TG) in BW–3, higher NEFA in BW–1, higher glucose (Glu) in BW–3, lower γ-glutamyl transferase (GGT) in BW–5, lower inorganic phosphate (iP) in BW–3, and higher body condition score (BCS) in BW–5 and BW–3 were significant compared with CON. Multiparous KET showed decreased cOC and uncarboxylated osteocalcin (ucOC) after parturition, which was lower than that in the CON group.

**Discussion:**

The blood parameters expressing different values between CON and KET in prepartum or early postpartum periods are presumed to show individual nutrition and health states, liver function, and overweight status. These parameters could be valuable indicators that can be used to prevent the occurrence of ketosis and improve management practices by recognizing these differences in ketotic cows before calving.

## Introduction

The periparturient period of dairy cows, before and after parturition, involves various risks associated with metabolic disorders. Ketosis is one of the most prevalent diseases causing large economic losses in terms of milk production, reproductivity, culling rate, and occurrence of other related diseases (1). When a cow begins lactation, an increase in energy demand for milk production and recovery and a decrease in dry matter intake induce a negative energy balance (NEB) in the early postpartum period (2). Under conditions of insufficient energy, lipolysis of adipose tissue releases non-esterified fatty acids (NEFA) into the blood, which the liver can take up and convert into additional energy (3). Although this metabolic pathway is a compensatory response to insufficient energy, the excessive mobilization of adipose tissue and uncontrolled production of ketone bodies can induce ketosis (4).

Metabolites derived from periparturient lipomobilization under the NEB status may have a lipotoxic effect on liver function (5). Energy deficiency also causes the loss of hormonal responsiveness (6), and the increase in circulating β-hydroxybutyrate (BHB) concentration in the early postpartum period can decrease the probability of pregnancy (7). As a cut-off point to define ketosis, BHB levels above 1.2–1.4 mmol/L in serum or plasma are used to diagnose subclinical ketosis, which is defined as an elevated concentration of BHB without clinical signs, whereas levels above 3.0 mmol/l are diagnosed as clinical ketosis (8, 9). Increased BHB concentration is an obvious risk factor for increasing the prevalence of postpartum disease, and several studies have suggested that circulating NEFA or BHB levels in the prepartum period or at parturition are predictors of the displaced abomasum (DA) (10, 11), retained placenta (12), and other postpartum diseases (13, 14). In addition, changes in blood biochemical parameters on the calving date have been suggested as indicators associated with postpartum increase in NEFA or BHB concentrations (15). The management of transition dairy cows can be challenging due to the various energy metabolism processes associated with ketosis. However, careful monitoring and analysis of blood indicators before calving can be highly beneficial in controlling the disease.

In the early lactation period, the production of colostrum and milk requires energy and a large amount of maternal Ca, and the consequent demand leads to a decrease in Ca concentration in the blood, which is a common factor in postpartum diseases (16). When Ca homeostasis is rigorously challenged, it affects energy metabolism as a result of overstimulation of lipolysis, reduction of feed intake, and suppression of immune functions (17, 18). Osteocalcin (OC) is a contextual factor that reflects the metabolic status of an individual or herd not only as a marker of bone growth and remodeling. OC is a protein hormone secreted by osteoblasts, and its carboxylated (cOC) form binds to Ca for bone mineralization (19, 20). Uncarboxylated OC (ucOC) regulates glucose and lipid metabolism by enhancing insulin secretion (19, 21). However, the precise mechanisms by which cOC and ucOC regulate glucose and lipid homeostasis remain unclear.

In this study, peripartum dairy cows with ketosis were defined and blood analysis results of both the prepartum and postpartum periods were compared retrospectively. Moreover, the OC concentrations in selected peripartum dairy cows were analyzed to evaluate the peripartum changes according to the ketosis occurrence.

## Materials and methods

### Animals

A total of 135 Holstein Friesian cows raised on a free-stall farm at Seoul National University, Pyeongchang, Republic of Korea, were used in this study. The cows were fed twice a day (Table 1) with a total mixed ration (TMR) and had free access to water. After parturition, participants voluntarily milked two or three times a day using a robotic milking system (VMS™, DeLaval, Tumba, Sweden). From April 2014 to December 2021, 210 parturitions of 114 primiparous and 96 multiparous cows were recorded. The primiparous cows had a mean age of 27.4 ± 5.0 months at parturition, and the multiparous cows had a mean age of 49.3 ± 12.8 (average ± S.D.) months at their 2nd–5th parities, with an average of 2.5 ± 0.8.

**Table 1:** Composition of hay, concentrate, and total mixed ration (TMR) fed to periparturient cows expressed as a percentage of the original feed substrate.

	Prepartum cow		Postpartum cow		
	Hay	Concentrate	Hay	Concentrate	TMR
Crude protein	10.06	18.83	10.06	22.8	15.27
Crude fat	2.45	3.42	2.45	5.54	4.32
Crude fiber	25.14	8.32	25.14	6.63	18.5
Crude ash	6.64	10.69	6.64	8.68	7.39
Calcium	0.39	1.71	0.39	1.71	0.874
Phosphorus	0.22	0.59	0.22	0.589	0.451
NE_L_ (Mcal/kg)	1.19	1.717	1.19	1.868	1.529
Feeding (kg/day)	6.0	3.5	6.0	milk yield × 0.28	20

The daily feeding amount for prepartum cows was determined by adding up the amounts of hay and concentrate. For postpartum cows, the amount of concentrate was adjusted based on their milk yield, and TMR was added to their diet. TMR, total mixed ration; NE_L_, net energy for lactation.

### Blood samples

Every 2 weeks during the periparturient period of cows, from approximately −60 d of expected parturition to 60 d of lactation to the maximum, coccygeal blood samples were collected in K_2_ EDTA and sodium heparin tubes and then stored in an insulated cooler box (2–8°C) until transportation. Due to the proximity of veins and arteries, some of the samples have been arterial blood draws. At the same time, the conditions of the fetus and uterus were confirmed in prepartum cows. Health status was examined in postpartum cows, and milk production and dry matter intake (DMI) were assessed. The body condition score (BCS) was also evaluated in both prepartum and postpartum cows. An injection of vitamin compound (Vigantol-E, Elanco Animal Health Incorporated, Indiana, United States) was administered once a month during the prepartum period. During the whole periparturient period, time points from 6 weeks before calving to 4 weeks after calving were selected and divided into five groups (BW–5, −6 to −5 weeks; BW–3, −4 to −3 weeks; BW–1, −2 to −1 weeks; BW1, 1 to 2 weeks; and BW3, 3 to 4 weeks of parturition).

### Blood analyses and group definition

The collected blood samples were transported to the laboratory of the hospital within 2 h, and complete blood count (CBC) tests were performed (Hemavet, Drew Scientific, Florida, USA). The blood in sodium heparin was centrifuged at 3,000 × *g* for 15 min, and the biochemical parameters of plasma were analyzed (BS-400, Mindray, Guangdong, China). The remaining plasma samples were stored at −80°C for further analysis. According to the plasma biochemistry results of BHB or NEFA in the postpartum period, the cows with either BHB ≥ 1.4 mmol/L or NEFA ≥0.7 mmol/L were defined as ketotic (KET, *n* = 116, 60 primiparous and 58 in multiparous) cows while those with lower levels were classified as non-ketotic (CON, *n* = 92, 54 primiparous and 38 multiparous) cows.

### Plasma analyses for osteocalcin

Cows were randomly selected within KET and CON groups, respectively, for the analysis of cOC (*n* = 16 in KET and *n* = 15 in CON) and ucOC (*n* = 17 in KET and *n* = 18 in CON) concentrations, and plasma samples from BW–1 and BW1 were used. The analysis was performed using cOC and ucOC ELISA kits (MK111 and MK118, Takara, Shiga, Japan), and the intra-and inter-assay coefficients of variation (CV) were 4.3 and 3.6% for cOC and 5.9 and 7.8% for ucOC, respectively.

### Statistical analyses

At each time point and for each blood parameter, an independent *t*-test was performed to compare the KET and CON cows using blood analysis data (SPSS Statistics 26, IBM, New York, United States). The Mann–Whitney test was used to detect differences when the data were not normally distributed by the Shapiro–Wilk test. To compare the results of OC concentrations, an independent *t*-test was performed between KET and CON at each time point, and a paired *t*-test or a Wilcoxon signed-rank test was used between BW–1 and BW1 to analyze parturition-related changes in each group.

## Results

### Hematological and plasma biochemical analyses in prepartum periods

The results of the CBC and the plasma biochemical analyses were presented from 6 weeks before (Tables 2–4 and Supplementary Table S1) to 4 weeks after (Tables 5, 6 and Supplementary Table S2) parturition in the primiparous and multiparous cows. Supplementary data in our study refers to the parameters that did not exhibit significant differences during the prepartum or postpartum period. In the prepartum period (Tables 2–4), primiparous KET showed lower numbers of lymphocytes (Lym) and red blood cells (RBC) in BW–5 (Table 2, *p* = 0.015 and 0.017, respectively) and Lym in BW–3 (Table 3, *p* = 0.024), while multiparous KET showed lower neutrophil (Neu) numbers in BW–5 (Table 2, *p* = 0.020). The concentration of hemoglobin (HGB) and the percentage of hematocrit (HCT) were higher in the prepartum KET, and the differences in HGB were significant in multiparous cows in BW–5 (Table 2, *p* = 0.023). The mean corpuscular volume (MCV) and mean corpuscular hemoglobin (MCH) were higher in the KET of both primiparous and multiparous cows, and these differences were observed throughout the prepartum period.

**Table 2:** Parameters of complete blood count (CBC), biochemistry analysis and BCS from −6 to −5 weeks of parturition (BW–5).

Prepartum	Parameters	Primiparous			Multiparous		
		CON (*n* = 54)	KET (*n* = 60)	*p* value	CON (*n* = 38)	KET (n = 58)	*p* value
BW–5	Neu, 10^3^/mm^3^	2.9 ± 0.8	2.8 ± 1.0	0.73	3.2 ± 1.0	2.7 ± 1.0	0.020
	Lym, 10^3^/mm^3^	3.9 ± 0.8	3.4 ± 0.8	0.015	3.3 ± 1.2	3.0 ± 0.9	0.37
	RBC, 10^6^/mm^3^	6.7 ± 0.8	6.4 ± 0.5	0.017	6.3 ± 0.5	6.3 ± 0.7	0.75
	HGB, g/dL	10.6 ± 1.5	10.6 ± 0.9	0.98	9.9 ± 1.0	10.4 ± 1.2	0.023
	MCV, fL	41.8 ± 4.0	43.0 ± 3.0	0.16	42.1 ± 3.3	44.2 ± 4.7	0.046
	MCH, pg	15.9 ± 2.2	16.7 ± 1.6	0.076	15.7 ± 1.8	16.7 ± 1.7	0.010
	TC, mg/dL	104.7 ± 20.0	105.2 ± 23.7	0.93	129.5 ± 43.4	111.4 ± 27.9	0.037
	TG, mg/dL	23.7 ± 6.0	21.1 ± 5.2	0.067	20.0 ± 6.4	21.8 ± 5.3	0.17
	NEFA, mmol/L	0.16 ± 0.12	0.20 ± 0.13	0.29	0.11 ± 0.09	0.14 ± 0.11	0.10
	Glu, mg/gL	71.0 ± 6.9	73.8 ± 6.7	0.068	67.3 ± 8.4	69.2 ± 7.6	0.28
	GGT, U/L	15.8 ± 3.5	16.2 ± 5.1	0.82	21.0 ± 6.9	19.3 ± 5.4	0.039
	iP, mg/dL	6.6 ± 0.7	6.7 ± 0.7	0.75	6.4 ± 0.8	6.4 ± 0.6	0.84
	BCS	3.46 ± 0.16	3.47 ± 0.18	0.71	3.36 ± 0.18	3.48 ± 0.21	0.010

The results are expressed as means ± standard deviations and p value. Neu, neutrophil; Lym, lymphocytes; RBC, red blood cell; HGB, hemoglobin; MCV, mean corpuscular volume; MCH, mean corpuscular hemoglobin; TC, cholesterol; TG, triglyceride; NEFA, non-esterified fatty acids; Glu, glucose; GGT, γ-glutamyl transferase; iP, inorganic phosphate; BCS, body condition score.

**Table 3:** Parameters of complete blood count (CBC), biochemistry analysis and BCS from −4 to −3 weeks of parturition (BW–3).

Prepartum	Parameters	Primiparous			Multiparous		
		CON (*n* = 54)	KET (*n* = 60)	*p* value	CON (*n* = 38)	KET (*n* = 58)	*p* value
BW–3	Neu, 10^3^/mm^3^	3.3 ± 0.9	3.1 ± 0.8	0.21	3.0 ± 0.8	2.9 ± 0.8	0.34
	Lym, 10^3^/mm^3^	3.8 ± 0.9	3.5 ± 1.6	0.024	3.2 ± 1.4	3.0 ± 0.8	0.91
	RBC, 10^6^/mm^3^	6.7 ± 0.6	6.5 ± 0.6	0.081	6.3 ± 0.4	6.2 ± 0.6	0.56
	HGB, g/dL	10.7 ± 1.2	10.8 ± 1.2	0.69	10.1 ± 1.0	10.4 ± 1.1	0.29
	MCV, fL	41.9 ± 3.8	42.7 ± 5.0	0.12	42.6 ± 3.5	44.4 ± 4.8	0.063
	MCH, pg	16.2 ± 2.1	16.8 ± 1.7	0.051	16.1 ± 1.6	16.7 ± 1.7	0.11
	TC, mg/dL	104.2 ± 19.8	100.1 ± 23.4	0.33	107.3 ± 33.2	105.7 ± 28.3	0.81
	TG, mg/dL	23.7 ± 7.0	21.9 ± 5.0	0.13	20.4 ± 4.7	23.9 ± 6.0	0.005
	NEFA, mmol/L	0.17 ± 0.11	0.25 ± 0.23	0.048	0.11 ± 0.08	0.15 ± 0.17	0.23
	Glu, mg/gL	71.9 ± 6.0	74.0 ± 6.0	0.089	67.3 ± 5.4	70.2 ± 6.2	0.033
	GGT, U/L	16.8 ± 4.7	16.7 ± 3.8	0.96	20.5 ± 4.7	19.1 ± 4.1	0.16
	iP, mg/dL	6.6 ± 1.0	6.6 ± 0.7	0.52	6.6 ± 0.8	6.2 ± 0.7	0.027
	BCS	3.47 ± 0.16	3.51 ± 0.17	0.40	3.40 ± 0.15	3.49 ± 0.18	0.011

The results are expressed as means ± standard deviations and *p* value. Neu, neutrophil; Lym, lymphocytes; RBC, red blood cell; HGB, hemoglobin; MCV, mean corpuscular volume; MCH, mean corpuscular hemoglobin; TC, cholesterol; TG, triglyceride; NEFA, non-esterified fatty acids; Glu, glucose; GGT, γ-glutamyl transferase; iP, inorganic phosphate; BCS, body condition score.

**Table 4:** Parameters of complete blood count (CBC), biochemistry analysis and BCS from −2 to −1 weeks of parturition (BW–1).

Prepartum	Parameters	Primiparous			Multiparous		
		CON (*n* = 54)	KET (*n* = 60)	*p* value	CON (*n* = 38)	KET (*n* = 58)	*p* value
BW–1	Neu, 10^3^/mm^3^	4.1 ± 1.4	4.0 ± 1.5	0.58	3.7 ± 1.0	3.7 ± 1.3	0.93
	Lym, 10^3^/mm^3^	3.5 ± 1.0	3.5 ± 1.3	0.98	3.1 ± 1.5	2.7 ± 0.9	0.62
	RBC, 10^6^/mm^3^	6.8 ± 0.7	6.7 ± 0.6	0.46	6.5 ± 0.6	6.4 ± 0.6	0.48
	HGB, g/dL	10.8 ± 1.4	11.1 ± 1.4	0.27	10.3 ± 0.9	10.7 ± 1.4	0.16
	MCV, fL	41.6 ± 3.6	43.5 ± 3.5	0.011	42.8 ± 3.8	45.0 ± 4.7	0.049
	MCH, pg	15.9 ± 1.9	16.6 ± 1.9	0.064	16.0 ± 1.8	16.7 ± 1.9	0.068
	TC, mg/dL	91.9 ± 22.0	86.7 ± 23.8	0.27	95.0 ± 31.0	90.5 ± 24.6	0.45
	TG, mg/dL	22.6 ± 7.0	21.6 ± 6.9	0.48	22.2 ± 7.3	22.6 ± 6.3	0.75
	NEFA, mmol/L	0.21 ± 0.12	0.28 ± 0.30	0.51	0.14 ± 0.11	0.19 ± 0.12	0.044
	Glu, mg/gL	70.4 ± 6.2	71.8 ± 9.0	0.38	66.7 ± 7.8	69.5 ± 6.9	0.074
	GGT, U/L	17.3 ± 4	16.9 ± 7.6	0.31	18.4 ± 5.4	18.6 ± 5.2	0.51
	iP, mg/dL	6.4 ± 1.4	6.5 ± 0.8	0.73	6.6 ± 0.8	6.5 ± 1.0	0.53
	BCS	3.47 ± 0.15	3.48 ± 0.17	0.76	3.43 ± 0.21	3.50 ± 0.20	0.085

The results are expressed as means ± standard deviations and *p* value. Neu, neutrophil; Lym, lymphocytes; RBC, red blood cell; HGB, hemoglobin; MCV, mean corpuscular volume; MCH, mean corpuscular hemoglobin; TC, cholesterol; TG, triglyceride; NEFA, non-esterified fatty acids; Glu, glucose; GGT, γ-glutamyl transferase; iP, inorganic phosphate; BCS, body condition score.

**Table 5:** Parameters of complete blood count (CBC) and biochemistry analysis from 1 to 2 weeks of parturition (BW1).

Postpartum	Parameters	Primiparous			Multiparous		
		CON (*n* = 54)	KET (*n* = 60)	*p* value	CON (*n* = 38)	KET (*n* = 58)	*p* value
BW1	WBC, 10^3^/mm^3^	7.2 ± 2.8	7.2 ± 2.6	0.76	6.6 ± 2.3	6.9 ± 2.3	0.52
	Neu, 10^3^/mm^3^	3.4 ± 2.4	3.6 ± 2.1	0.52	3.2 ± 1.5	3.7 ± 1.9	0.23
	Lym, 10^3^/mm^3^	2.9 ± 0.8	2.8 ± 0.8	0.47	3.0 ± 1.2	2.5 ± 0.8	0.072
	HGB, g/dL	10.6 ± 1.5	10.6 ± 1.4	0.96	10.1 ± 1.1	10.8 ± 1.1	0.002
	HCT, %	27.4 ± 2.9	28.1 ± 3.6	0.43	26.4 ± 2.6	28.4 ± 2.9	0.001
	MCV, fL	41.9 ± 3.6	43.2 ± 3.3	0.058	42.7 ± 3.4	44.9 ± 4.6	0.044
	MCH, pg	16.2 ± 1.8	16.3 ± 1.8	0.48	16.4 ± 1.7	17.1 ± 1.2	0.018
	PLT, 10^3^/mm^3^	327.1 ± 129.3	316.2 ± 166.0	0.70	337.2 ± 100.1	286.9 ± 105.9	0.047
	TC, mg/dL	93.7 ± 24.6	78.0 ± 26.3	0.001	91.8 ± 34.8	87.5 ± 40.4	0.29
	TG, mg/dL	10.7 ± 3.7	12.2 ± 4.0	0.014	8.6 ± 2.0	10.4 ± 3.2	0.001
	BHB, mmol/L	0.69 ± 0.21	1.23 ± 0.87	<0.001	0.63 ± 0.17	1.28 ± 0.77	<0.001
	NEFA, mmol/L	0.42 ± 0.15	0.95 ± 0.38	<0.001	0.33 ± 0.15	0.79 ± 0.40	<0.001
	Glu, mg/gL	69.6 ± 14.9	64.5 ± 16.7	0.038	63.0 ± 12.5	59.3 ± 15.5	0.23
	BUN, mg/dL	12.7 ± 3.1	12.0 ± 2.9	0.24	13.0 ± 3.4	12.9 ± 3.4	0.96
	AST, U/L	90.3 ± 22.0	125.9 ± 67.9	<0.001	86.3 ± 17.5	116.0 ± 80.7	0.007
	GGT, U/L	20.4 ± 5.5	21.7 ± 8.4	0.60	20.5 ± 6.2	22.8 ± 12.7	0.81
	LDH, U/L	2139.8 ± 419.1	2358.9 ± 546.3	<0.001	1847.6 ± 250.4	2061.9 ± 616.4	0.094
	CK, U/L	219.1 ± 165	407.7 ± 531	<0.001	184.8 ± 176.0	236.6 ± 194.2	0.013
	ALP, U/L	67.2 ± 19.0	73.2 ± 25.7	0.22	48.9 ± 16.9	52.1 ± 17.7	0.37
	Ca, mg/dL	8.9 ± 0.7	8.8 ± 0.6	0.17	8.9 ± 0.6	8.6 ± 0.8	0.020

The results are expressed as means ± standard deviations and *p* value. WBC, white blood cell; Neu, neutrophil; Lym, lymphocytes; HGB, hemoglobin; HCT, hematocrit; MCV, mean corpuscular volume; MCH, mean corpuscular hemoglobin; PLT, platelet; TC, cholesterol; TG, triglyceride; BHB, β-hydroxybutyrate; NEFA, non-esterified fatty acids; Glu, glucose; BUN, blood urea nitrogen; AST, aspartate aminotransferase; GGT, γ-glutamyl transferase; LDH, lactate dehydrogenase; CK, creatine kinase; ALP, alkaline phosphatase; Ca, total calcium.

**Table 6:** Parameters of complete blood count (CBC) and biochemistry analysis from 3 to 4 weeks of parturition (BW3).

Postpartum	Parameters	Primiparous			Multiparous		
		CON (*n* = 54)	KET (*n* = 60)	*p* value	CON (*n* = 38)	KET (*n* = 58)	*p* value
BW3	WBC, 10^3^/mm^3^	9.1 ± 3.1	7.3 ± 2.3	0.003	7.5 ± 1.8	6.7 ± 2.0	0.065
	Neu, 10^3^/mm^3^	4.9 ± 2.6	3.6 ± 1.7	0.007	3.8 ± 1.3	3.6 ± 1.7	0.26
	Lym, 10^3^/mm^3^	3.2 ± 0.9	3.2 ± 1.2	0.57	3.1 ± 1.2	2.6 ± 0.6	0.019
	HGB, g/dL	9.5 ± 1.1	9.4 ± 1.4	0.65	9.4 ± 0.9	9.4 ± 1.1	0.93
	HCT, %	24.6 ± 2.1	24.7 ± 3.3	0.78	24.3 ± 1.9	24.8 ± 2.5	0.32
	MCV, fL	40.8 ± 3.7	41.7 ± 2.9	0.15	41.8 ± 3.2	44.0 ± 4.5	0.026
	MCH, pg	15.9 ± 2.0	15.9 ± 1.8	0.83	16.2 ± 1.7	16.6 ± 2.0	0.34
	PLT, 10^3^/mm^3^	407.6 ± 130.6	438.9 ± 164.9	0.29	369.8 ± 103.0	376.2 ± 129.5	0.82
	TC, mg/dL	144.1 ± 33.5	131.5 ± 37.5	0.074	160.8 ± 36.3	149.8 ± 44.9	0.26
	TG, mg/dL	10.3 ± 2.1	11.0 ± 3.4	0.51	9.1 ± 2.3	10.5 ± 2.5	0.014
	BHB, mmol/L	0.72 ± 0.23	1.45 ± 0.88	<0.001	0.82 ± 0.21	1.63 ± 1.17	<0.001
	NEFA, mmol/L	0.34 ± 0.13	0.67 ± 0.43	<0.001	0.28 ± 0.14	0.53 ± 0.25	<0.001
	Glu, mg/gL	63.8 ± 10.4	59.3 ± 14.5	0.069	57.8 ± 7.0	52.7 ± 10.1	0.008
	BUN, mg/dL	13.8 ± 3.6	12.1 ± 3.7	0.050	13.4 ± 3.2	12.8 ± 3.2	0.45
	AST, U/L	77.0 ± 12.8	107.7 ± 55.1	<0.001	86.6 ± 15.4	101.4 ± 28.5	0.003
	GGT, U/L	24.1 ± 19.0	32.2 ± 22.2	<0.001	23.9 ± 7.2	35.8 ± 57.0	0.13
	LDH, U/L	2045.6 ± 375.4	2534.8 ± 682.1	<0.001	1982.3 ± 289.1	2274.2 ± 620.5	0.005
	CK, U/L	244.9 ± 199.2	283.8 ± 267.3	0.55	170.9 ± 39.6	207.1 ± 126.6	0.44
	ALP, U/L	57.5 ± 18.6	59.8 ± 17.3	0.44	37.9 ± 7.4	44.9 ± 18.2	0.048
	Ca, mg/dL	9.1 ± 0.6	9.1 ± 0.7	0.87	9.2 ± 0.5	9.1 ± 0.7	0.47

The results are expressed as means ± standard deviations and *p* value. WBC, white blood cell; Neu, neutrophil; Lym, lymphocytes; HGB, hemoglobin; HCT, hematocrit; MCV, mean corpuscular volume; MCH, mean corpuscular hemoglobin; PLT, platelet; TC, cholesterol; TG, triglyceride; BHB, β-hydroxybutyrate; NEFA, non-esterified fatty acids; Glu, glucose; BUN, blood urea nitrogen; AST, aspartate aminotransferase; GGT, γ-glutamyl transferase; LDH, lactate dehydrogenase; CK, creatine kinase; ALP, alkaline phosphatase; Ca, total calcium.

Among the biochemical parameters, the plasma concentrations of total cholesterol (TC), triglyceride (TG), NEFA, and glucose (Glu) were different depending on the parity and parturition. TC gradually decreased until BW1 in all groups except primiparous CON, but there was no statistical analysis performed (Tables 2–4). The multiparous KET cows showed lower TC from BW–5 (Table 2, *p* = 0.037) and higher TG from BW–3 (Table 3, *p* = 0.005) compared with CON. There were prepartum increases in NEFA in primiparous (Table 3, *p* = 0.048) and multiparous (Table 4, *p* = 0.044) KET cows, and the Glu levels were higher in KET throughout the entire prepartum period in both primiparous and multiparous cows. The multiparous KET group showed lower γ-glutamyl transferase (GGT) levels (Table 2, *p* = 0.039) and lower inorganic phosphate (iP) levels (Table 3, *p* = 0.027). Prepartum BCS was higher in KET cows, but this difference was not statistically significant in primiparous cows (Tables 2, 3).

### Hematological and plasma biochemical analyses in postpartum periods

In the postpartum period (Tables 5, 6), lower numbers of white blood cells (WBC), Neu, and Lym were present in primiparous or multiparous KET in BW3 (Table 6). For the other hematological parameters, including HGB, HCT, MCV, and MCH, KET showed higher levels than CON, especially in multiparous cows in BW1 (Table 5). The multiparous KET group had a lower platelet count (PLT) than the CON group in BW1 (Table 5, *p* = 0.047).

Among the biochemical parameters, KET cows showed lower TC and higher TG compared with CON (Tables 5, 6), and the differences were observed in both primiparous and multiparous cows. BHB and NEFA were higher in both primiparous and multiparous KET, which is consistent with the definition of ketosis, and they showed lower Glu levels (Tables 5, 6). The lower level of blood urea nitrogen (BUN) following postpartum ketosis in primiparous cows was weakly significant (Table 6, *p* = 0.050). The plasma levels of aspartate aminotransferase (AST), GGT, lactate dehydrogenase (LDH), creatine kinase (CK), and alkaline phosphatase (ALP) were higher in the KET group in primiparous or multiparous cows (Tables 5, 6), and the lower Ca was present in multiparous cows in BW1 (Table 5).

### Osteocalcin analyses and ketosis

**Figure 1:**
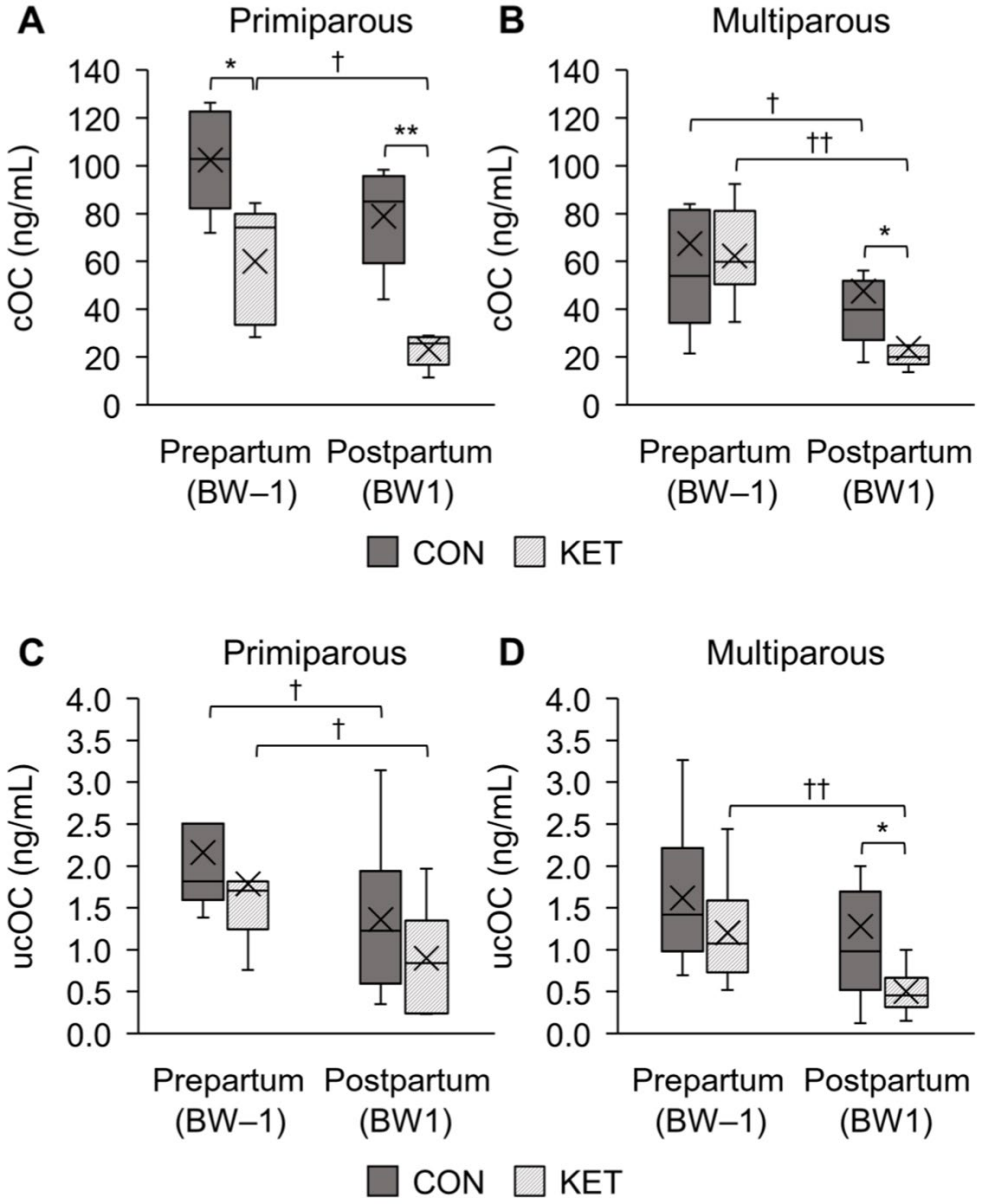
Box-and-whisker plot comparison of plasma concentrations of osteocalcin (OC) between healthy cows (CON) and ketotic cows (KET) in prepartum (BW–1; −2 to −1 weeks of parturition) and postpartum (BW1; 1 to 2 weeks of parturition). The carboxylated osteocalcin (cOC) in primiparous **(A)** and multiparous **(B)** cows are presented, and the uncarboxylated osteocalcin (ucOC) is also analyzed in primiparous **(C)** and multiparous **(D)** cows. Statistical comparisons were performed, and the significances between CON and KET (^*^ *p* < 0.05, ^**^ *p* < 0.01) and between BW–1 and BW1 (^†^ *p* < 0.05, ^††^ *p* < 0.01) were presented.

The cOC and ucOC levels of primiparous and multiparous cows are presented in Figure 1, and each subfigure shows the average levels with distribution and the significance according to ketosis occurrence (CON and KET) and parturition (BW–1 and BW1). For the analysis of cOC level, the plasma samples of the primiparous CON (*n* = 5, 26.8 ± 4.3; the mean ± standard deviation of monthly age at parturition), primiparous KET (*n* = 5, 34.0 ± 4.7), multiparous CON (*n* = 10, 52.3 ± 12.5), and multiparous KET (*n* = 11, 54.7 ± 12.6) were selected. For the ucOC analysis, primiparous CON (*n* = 6, 26.5 ± 3.9), primiparous KET (*n* = 7, 32.4 ± 4.8), multiparous CON (*n* = 12, 50.5 ± 12.2), and multiparous KET (*n* = 10, 54.0 ± 13.0) were selected. The average age at parturition was statistically different between the primiparous CON and KET groups for both cOC and ucOC (*p* = 0.036 and 0.035, respectively).

Statistical comparisons between CON and KET were performed for cOC and ucOC, and in BW–1, only prepartum primiparous cows for cOC showed statistical differences (Figure 1A *p* = 0.021). The postpartum cows (BW1) showed statistical differences for the primiparous cOC (Figure 1A *p* = 0.001), multiparous cOC (Figure 1B *p* = 0.016), and multiparous ucOC (Figure 1D *p* = 0.017). As a result of the postpartum decrease in OC, differences were observed when we compared the prepartum and postpartum cows in the primiparous cOC (Figure 1A *p* = 0.14 and 0.043 in CON and KET, respectively), multiparous cOC (Figure 1B *p* = 0.017 and 0.003), primiparous ucOC (Figure 1C *p* = 0.028 both), and multiparous ucOC (Figure 1D *p* = 0.071 and 0.005).

## Discussion

In the present study, we aimed to evaluate the differences in the concentrations of hematological and biochemical parameters and osteocalcin related to ketosis between CON and KET during the periparturient period and to screen for prepartum or early postpartum parameters that could predict the occurrence of ketosis. The metabolic changes in KET in the postpartum period reflected the obvious differences in the blood analysis compared with the CON group, as expected. In the prepartum period (BW–5, BW–3, and BW–1) and the early postpartum period (BW1), we found several parameters, implying potential as a detection for ketosis occurrence, which is defined as an increase in BHB or NEFA in the postpartum period. Each parameter showed a different significance in primiparous and multiparous cows. In primiparous cows, Lym, RBC, MCV, and NEFA had significantly different values between CON and KET at least one stage of the test period inthe prepartum period, but the differences were limited in fewer parameters and periods compared with multiparous cows. In multiparous cows, higher values of HGB, MCV, MCH, TG, NEFA, Glu, and BCS, and lower values of Neu, TC, GGT, and iP were observed in the prepartum period. In addition, the decreases in cOC and ucOC levels of the multiparous KET were observed in the periods from BW–1 to BW1, which were more significant and lowered than CON, whereas the prepartum and postpartum differences were significant only for the cOC levels in the primiparous cows.

Increases in Neu and Lym counts mainly indicate systemic inflammation or other pathological symptoms. Hammon et al. (22) presented a correlation between NEFA concentration and Neu activity. In the peripartum period, the number of circulating Neu or activity tends to decrease due to mammary involution, recruitment to the peripheral tissues, or hypocalcemia (23). Conversely, increased numbers of Neu and insulin resistance are related to adipose tissue in overweight individuals (24), and stressful conditions, including ketosis, abomasal displacement, and dystocia, can induce Neu in cows (25). A decrease in Lym count is also related to insulin resistance (26), and both NEFA and BHB inhibit the development and function of Lym (27, 28). It is uncertain which factor gave rise to differences between the CON and the KET groups since evidence of an infection or changes in physical symptoms were not detected in the prepartum cows.

The primiparous KET group in the early prepartum period showed a lower number of RBC than the CON group, but we could not directly relate the lower RBC numbers to anemia. Rather, we observed a slight increase in the concentrations of HGB, MCV, and MCH, especially in the multiparous KET in both the prepartum and postpartum periods. The increase in MCV and MCH suggests macrocytosis, which is mostly found in cirrhotic patients with alcoholic or non-alcoholic disease (29), and the early postpartum values were consistent with a previous report (15). However, in the prepartum period, no noticeable increases in biochemical parameters related to liver damage or vitamin B12/folate deficiency were observed (30). Kamruzzaman (31) evaluated anemia and HGB levels according to BMI scores in humans thus it would also be relevant to evaluate both BCS and HGB-related parameters in the present study. In a study on a ketogenic diet in rats, the MCV and MCH levels decreased with acidosis (32), implying that the effects of ketone bodies are not superior to those in this study. Low platelet numbers can also be attributed to liver disease or folate deficiency (33, 34), and the lower PLT in KET compared with CON in the present study needs to be evaluated together with additional analysis for postpartum liver function or nutrition.

As a predominant factor, which indicates NEB and lipolysis (35), the concentration of NEFA increased just before parturition and was significant in primiparous and multiparous cows. Cholesterol concentration is one of the parameters used to predict fatty liver and other postpartum diseases, with a ratio to NEFA in cows (36, 37). In a study by Tessari et al. (35), fatty acids were evaluated according to lipid classes, and healthy cows with lower NEFA had higher circulating cholesterol esters (CEs) with higher lecithin cholesterol acyl-transferase (LCAT) activity in the early postpartum period. The negative correlation between NEFA and blood cholesterol was also explained by an accumulation of TG in the liver, followed by a reduction in DMI (38). Furthermore, the low cholesterol concentration in cows with fatty liver (39), can be a basis for presuming the prepartum health status of multiparous KET in the present study. High TG concentrations in the postpartum KET showed contrary results to those of a previous study which suggested a negative correlation between NEFA and plasma TG levels (40). Oikawa et al. (41), however, suggested a positive correlation between NEFA and VLDL-TG concentrations in the serum of primiparous cows rather than multiparous cows, and in the analysis of plasma fatty acids (FA) related to TG (41), the total FA increased in hyperketonemic cows. In addition, they suggested that the ability to synthesize VLDL differs depending on parity, which supports the prepartum TG levels of primiparous and multiparous cows in the present study. Although cattle have a limited ability to export TG, and their blood levels do not accurately reflect liver accumulation (42), increased TG is one of the main parameters used to evaluate liver function in cows (43). Lipid metabolism-related blood parameters can be complicated by parities, the time point of sampling or analyses, fatty liver, nutrition states, and parturition stress; however, the differences between CON and KET in the present study exhibited potential as a preventative marker of ketosis. As indicators of liver and muscle damage (15, 44, 45), higher levels of AST, GGT, LDH, CK, and ALP in KET reflect the postpartum liver function for sudden energy consumption, but the reason for the prepartum difference in GGT is not clear. Calcium concentration after parturition is related to disturbances in energy metabolism (18), and it is significantly lower immediately after parturition in multiparous cows.

The role of skeleton-derived osteocalcin in energy metabolism has been confirmed in knockout mice expressing obesity and hyperglycemia, with insulin insufficiency and resistance (19). Both cOC and ucOC in mice are associated with decreased fat mass, increased glucose transport, and improved insulin sensitivity *in vivo* (46) and *in vitro* (47). In non-periparturient cows, an age-related decrease was observed as parity increased (48), and their findings support the difference in prepartum or postpartum cOC levels between CON and KET in primiparous cows in the present study. The age-related decrease in ucOC was less significant than that in cOC. The concentration of ucOC in postpartum multiparous cows (Figure 1D) could be explained by the low ucOC levels and low insulin sensitivity in postpartum cows (49). In addition, the changes in cOC or ucOC levels between BW–1 and BW1 and the different postpartum levels between CON and KET suggest that both cOC and ucOC are involved in energy metabolism related to postpartum ketosis. Thus, the low levels of OC (both cOC and ucOC), which decreased abruptly in BW1, provide a rationale for managing transition cows at high risk for ketosis in the early postpartum period. Exogenous calcitriol administration increases the plasma concentrations of ucOC and cOC in cattle (50), and in the present study, cows were injected with vitamin D intramuscularly before parturition at a 4-week interval. The effect of vitamin D injection could not be analyzed due to the uncontrolled administration date, however, it is possible that controlled administration of a variety of forms of vitamin D before parturition prevents ketosis through increased plasma concentrations of ucOC and cOC. To elucidate these, further investigations are needed on the effects of OC changes with various health states and the mechanisms by which OC is involved in energy metabolism.

The inclusion of a comparison between primiparous and multiparous cows provides valuable information for understanding the differences in ketosis incidence and blood test values between these two groups. It is noteworthy that high NEFA levels were observed in both primiparous and multiparous cows before parturition, whereas high BCS, low TC, and high TG levels were only significant in multiparous cows. Additionally, low TC and Glu levels in cows with ketosis after parturition appeared earlier in primiparous cows, and there was no significant difference in Ca levels between the two groups. The differences in blood test values may be attributed to the differences in energy metabolism and nutritional requirements between these two groups. Primiparous cows produce less milk than multiparous cows, and the imbalance of energy metabolism before parturition assessed by blood biochemistry results was not noticeable. Therefore, management strategies to prevent and manage ketosis should take into account the specific needs and characteristics of both primiparous and multiparous cows. In primiparous cows, the rumen filling score (41) can be a useful tool for assessing DMI and ensuring that cows are receiving an adequate supply of nutrients to support their energy requirements.

In conclusion, several hematologic and biochemical parameters were significantly different in ketotic cows in both the prepartum and postpartum periods. The increased levels of MCV and MCH in the prepartum period suggested the occurrence of ketosis related to hepatic metabolism, which appeared earlier in the prepartum period, especially in multiparous cows. Among the parameters of energy metabolism, low TC and high BCS and TG in KET cows before parturition suggested impaired hepatic lipid metabolism and increased insulin resistance related to ketosis, which appeared prominently in multiparous cows. In the OC analyses, the largely decreased cOC and ucOC after parturition in KET indicated that OC is related to energy metabolism and ketosis occurrence in periparturient cows. To identify the metabolic parameters of ketosis occurrence, this study was conducted based only on ketosis, without considering other postpartum diseases in cows. Since only blood analysis was performed every 2 weeks, comparative analysis with other indicators, such as TG concentration in the liver, is limited, and it might be less subdivided in inferring change than a daily analysis. In this study, however, it was able to identify differences in blood analysis values between CON and KET for each evaluation period before and after parturition. The significantly different levels of prepartum blood parameters and the postpartum changes in osteocalcin level can be used to detect cows with a high risk of ketosis, and it helps improve cattle productivity and understand metabolic processes related to ketosis.

## Data availability statement

The original contributions presented in the study are included in the article/Supplementary material, further inquiries can be directed to the corresponding author.

## Ethics statement

Ethical review and approval were not required for the animal study because blood samples were collected and analyzed at Farm Animal Medical Training Hospital for the purpose of diagnosis and treatment.

## Author contributions

The conceptualization, validation, and investigation of this study was done by WC, YR, and DaK. The data curation and statistical analysis was developed by WC and DaK, and the research resources were provided by WC, YR, EC, LH, DoK, SK, IY, and DaK. WC wrote the original draft, and review and editing were done by WC and DaK. All authors contributed to the article and approved the submitted version.

## Funding

This study was partially supported by the Research Institute for Veterinary Science, Seoul National University.

## Conflict of interest

The authors declare that the research was conducted in the absence of any commercial or financial relationships that could be construed as a potential conflict of interest.

## Publisher’s note

All claims expressed in this article are solely those of the authors and do not necessarily represent those of their affiliated organizations, or those of the publisher, the editors and the reviewers. Any product that may be evaluated in this article, or claim that may be made by its manufacturer, is not guaranteed or endorsed by the publisher.
