# Supplementary material for: Comparison of prepartum blood parameters in dairy cows with postpartum ketosis and new risk prediction candidates
Source: Front Vet Sci. 2023 May 11;10:1161596. doi: 10.3389/fvets.2023.1161596 (PMC10213766; doi:10.3389/fvets.2023.1161596)
Supplement: Supplementary file 1 [file Table_1.DOCX]

Supplementary Table S1. Parameters of complete blood count (CBC) and biochemistry analysis in prepartum periods. In comparing CON and KET, parameters with *p* > 0.05 were presented as supplementary data, and the results are expressed as means ± standard deviations and P value.

| Prepartum | Parameters | Primiparous | | |  | Multiparous | | |
| --- | --- | --- | --- | --- | --- | --- | --- | --- |
|  |  | CON | KET | P value |  | CON | KET | P value |
| BW–5 | WBC, 10^3^/mm^3^ | 7.6 ± 1.6 | 7.3 ± 1.4 | 0.31 |  | 7.0 ± 1.6 | 6.4 ± 1.7 | 0.14 |
|  | HCT, % | 28.0 ± 3.2 | 27.4 ± 2.4 | 0.31 |  | 26.5 ± 2.9 | 27.5 ± 2.9 | 0.12 |
|  | MCHC, g/dL | 38.0 ± 4.4 | 38.9 ± 3.5 | 0.44 |  | 37.4 ± 4.1 | 38.1 ± 4.2 | 0.46 |
|  | PLT, 10^3^/mm^3^ | 219.8 ± 113.2 | 247.6 ± 92.1 | 0.26 |  | 270.5 ± 85.1 | 235.7 ± 96.2 | 0.10 |
|  | TP, g/dL | 7.9 ± 0.7 | 7.9 ± 0.8 | 0.99 |  | 8.5 ± 0.7 | 8.6 ± 0.7 | 0.84 |
|  | Alb, g/dL | 3.9 ± 0.2 | 3.9 ± 0.2 | 0.27 |  | 3.9 ± 0.2 | 4.0 ± 0.3 | 0.56 |
|  | Glb, g/dL | 3.9 ± 0.7 | 3.9 ± 0.7 | 0.87 |  | 4.7 ± 0.7 | 4.6 ± 0.7 | 0.82 |
|  | BHB, mmol/L | 0.53 ± 0.13 | 0.55 ± 0.13 | 0.49 |  | 0.57 ± 0.17 | 0.56 ± 0.16 | 0.74 |
|  | Crea, mg/dL | 1.4 ± 0.2 | 1.4 ± 0.2 | 0.38 |  | 1.3 ± 0.2 | 1.3 ± 0.2 | 0.61 |
|  | BUN, mg/dL | 9.1 ± 3.6 | 10.4 ± 4 | 0.15 |  | 9.8 ± 4.4 | 11.4 ± 4.1 | 0.08 |
|  | AST, U/L | 61.8 ± 9.9 | 64.5 ± 25.6 | 0.31 |  | 68.3 ± 31.1 | 63.1 ± 17.6 | 0.59 |
|  | LDH, U/L | 1931.4 ± 341.6 | 2031.3 ± 376.8 | 0.25 |  | 1861.9 ± 312.5 | 1776.7 ± 271.9 | 0.19 |
|  | CK, U/L | 271.9 ± 383.4 | 223.2 ± 328.0 | 0.61 |  | 208.9 ± 219.3 | 169.3 ± 151.9 | 0.70 |
|  | ALP, U/L | 82.0 ± 24.5 | 79.4 ± 26.7 | 0.75 |  | 58.4 ± 24.4 | 52.5 ± 21.7 | 0.18 |
|  | Ca, mg/dL | 9.3 ± 0.5 | 9.2 ± 0.5 | 0.35 |  | 9.0 ± 0.4 | 9.0 ± 0.6 | 0.93 |
| BW–3 | WBC, 10^3^/mm^3^ | 8.0 ± 1.4 | 7.8 ± 1.9 | 0.22 |  | 7.0 ± 1.9 | 6.7 ± 1.3 | 0.69 |
|  | HCT, % | 27.9 ± 2.6 | 27.5 ± 3.5 | 0.54 |  | 26.8 ± 2.3 | 27.5 ± 2.6 | 0.17 |
|  | MCHC, g/dL | 38.6 ± 4.0 | 40.2 ± 9.2 | 0.45 |  | 38.0 ± 4.2 | 37.8 ± 3.7 | 0.84 |
|  | PLT, 10^3^/mm^3^ | 261.6 ± 108.7 | 265.3 ± 97.9 | 0.92 |  | 251.8 ± 89.8 | 248.7 ± 91.9 | 0.88 |
|  | TP, g/dL | 7.7 ± 0.7 | 7.8 ± 0.8 | 0.76 |  | 8.4 ± 0.8 | 8.2 ± 0.7 | 0.64 |
|  | Alb, g/dL | 3.9 ± 0.2 | 3.9 ± 0.2 | 0.11 |  | 3.9 ± 0.2 | 3.9 ± 0.3 | 0.35 |
|  | Glb, g/dL | 3.8 ± 0.67 | 3.8 ± 0.7 | 0.93 |  | 4.5 ± 0.8 | 4.3 ± 0.8 | 0.30 |
|  | BHB, mmol/L | 0.55 ± 0.14 | 0.58 ± 0.16 | 0.50 |  | 0.61 ± 0.17 | 0.57 ± 0.17 | 0.25 |
|  | Crea, mg/dL | 1.4 ± 0.2 | 1.3 ± 0.2 | 0.15 |  | 1.3 ± 0.2 | 1.3 ± 0.2 | 0.37 |
|  | BUN, mg/dL | 11.0 ± 3.7 | 11.3 ± 3.4 | 0.74 |  | 10.1 ± 4.2 | 12.0 ± 4.5 | 0.06 |
|  | AST, U/L | 62.4 ± 11.7 | 58.9 ± 14.1 | 0.06 |  | 61.5 ± 13.1 | 60.8 ± 10.5 | 0.97 |
|  | LDH, U/L | 1920.3 ± 405.7 | 1912.4 ± 252.2 | 0.63 |  | 1747.3 ± 216.4 | 1721.7 ± 256.1 | 0.58 |
|  | CK, U/L | 323.2 ± 547.2 | 203.3 ± 218.7 | 0.24 |  | 150.4 ± 119.6 | 158.4 ± 174.1 | 0.53 |
|  | ALP, U/L | 77.9 ± 23.3 | 78.8 ± 25.4 | 0.94 |  | 52.3 ± 23.2 | 52.5 ± 21.1 | 0.97 |
|  | Ca, mg/dL | 9.3 ± 0.5 | 9.1 ± 0.5 | 0.16 |  | 9.1 ± 0.4 | 9.1 ± 0.5 | 0.86 |
| BW–1 | WBC, 10^3^/mm^3^ | 8.6 ± 2.1 | 8.4 ± 2.0 | 0.65 |  | 7.55 ± 2.16 | 7.32 ± 1.77 | 0.60 |
|  | HCT, % | 28.2 ± 2.7 | 29.2 ± 3.1 | 0.11 |  | 27.7 ± 2.8 | 28.7 ± 3.0 | 0.08 |
|  | MCHC, g/dL | 38.5 ± 4.3 | 38.3 ± 3.9 | 0.53 |  | 37.4 ± 3.7 | 37.3 ± 3.7 | 0.92 |
|  | PLT, 10^3^/mm^3^ | 262.0 ± 110.1 | 282.0 ± 158.0 | 0.48 |  | 263.1 ± 101.8 | 250.5 ± 103.7 | 0.25 |
|  | TP, g/dL | 7.3 ± 0.8 | 7.2 ± 0.9 | 0.16 |  | 7.8 ± 0.9 | 7.8 ± 0.8 | 0.87 |
|  | Alb, g/dL | 3.8 ± 0.2 | 3.7 ± 0.3 | 0.17 |  | 3.9 ± 0.2 | 3.9 ± 0.3 | 0.76 |
|  | Glb, g/dL | 3.5 ± 0.8 | 3.5 ± 0.8 | 0.78 |  | 3.9 ± 0.9 | 4.0 ± 0.7 | 0.50 |
|  | BHB, mmol/L | 0.53 ± 0.12 | 0.70 ± 0.83 | 0.10 |  | 0.61 ± 0.17 | 0.65 ± 0.38 | 0.53 |
|  | Crea, mg/dL | 1.4 ± 0.2 | 1.3 ± 0.2 | 0.09 |  | 1.35 ± 0.24 | 1.35 ± 0.29 | 0.99 |
|  | BUN, mg/dL | 12.7 ± 5.0 | 12.1 ± 3.7 | 0.54 |  | 10.6 ± 4.2 | 11.9 ± 5.2 | 0.21 |
|  | AST, U/L | 59.6 ± 11.1 | 64.9 ± 41.9 | 0.98 |  | 64.5 ± 14.9 | 59.0 ± 10.1 | 0.21 |
|  | LDH, U/L | 1795.5 ± 290.4 | 1845.3 ± 409.5 | 0.56 |  | 1699.4 ± 214.7 | 1666.5 ± 268.9 | 0.55 |
|  | CK, U/L | 225.3 ± 375.5 | 280.0 ± 582.4 | 0.49 |  | 172.0 ± 219.2 | 136.6 ± 152.6 | 0.81 |
|  | ALP, U/L | 85.2 ± 21.8 | 79.4 ± 22.8 | 0.22 |  | 60.6 ± 25.7 | 54.8 ± 19.0 | 0.28 |
|  | Ca, mg/dL | 9.0 ± 0.5 | 9.0 ± 0.5 | 0.90 |  | 9.0 ± 0.4 | 9.0 ± 0.5 | 0.90 |

WBC; white blood cell, HCT; hematocrit, MCHC; mean corpuscular hemoglobin concentration, PLT; platelet, TP; total protein, Alb; albumin, Glb; globulin, BHB; β-hydroxybutyrate, Crea; creatinine, BUN; blood urea nitrogen, AST; aspartate aminotransferase, LDH; lactate dehydrogenase, CK; creatine kinase, ALP; alkaline phosphatase, Ca; total calcium

Supplementary Table S2. Parameters of complete blood count (CBC), biochemistry analysis and BCS in postpartum periods. In comparing CON and KET, parameters with *p* > 0.05 were presented as supplementary data, and the results are expressed as means ± standard deviations and P value.

| Postpartum | Parameters | Primiparous | | |  | Multiparous | | |
| --- | --- | --- | --- | --- | --- | --- | --- | --- |
|  |  | CON | KET | P value |  | CON | KET | P value |
| BW1 | RBC, 10^6^/mm^3^ | 6.6 ± 0.8 | 6.5 ± 0.8 | 0.54 |  | 6.2 ± 0.6 | 6.4 ± 0.6 | 0.21 |
|  | MCHC, g/dL | 38.8 ± 4.0 | 37.9 ± 4.1 | 0.17 |  | 38.4 ± 3.5 | 38.3 ± 3.4 | 0.82 |
|  | TP, g/dL | 7.9 ± 0.8 | 7.8 ± 0.7 | 0.48 |  | 7.9 ± 0.8 | 8.0 ± 0.7 | 0.72 |
|  | Alb, g/dL | 3.7 ± 0.3 | 3.7 ± 0.3 | 0.86 |  | 3.8 ± 0.3 | 3.8 ± 0.3 | 0.39 |
|  | Glb, g/dL | 4.2 ± 0.7 | 4.1 ± 0.7 | 0.37 |  | 4.1 ± 0.7 | 4.1 ± 0.6 | 0.98 |
|  | Crea, mg/dL | 1.1 ± 0.2 | 1.1 ± 0.2 | 0.74 |  | 1.1 ± 0.2 | 1.2 ± 0.2 | 0.27 |
|  | iP, mg/dL | 5.9 ± 1.3 | 6.0 ± 1.1 | 0.57 |  | 5.9 ± 1.1 | 5.7 ± 1.5 | 0.48 |
|  | BCS | 3.4 ± 0.2 | 3.4 ± 0.2 | 0.55 |  | 3.3 ± 0.2 | 3.3 ± 0.2 | 0.56 |
| BW3 | RBC, 10^6^/mm^3^ | 6.1 ± 0.6 | 5.9 ± 0.7 | 0.31 |  | 5.8 ± 0.6 | 5.7 ± 0.5 | 0.18 |
|  | MCHC, g/dL | 39.0 ± 4.5 | 38.2 ± 4.1 | 0.24 |  | 38.8 ± 3.7 | 37.9 ± 4.3 | 0.37 |
|  | TP, g/dL | 8.6 ± 0.7 | 8.5 ± 0.7 | 0.63 |  | 8.5 ± 0.9 | 8.6 ± 0.7 | 0.32 |
|  | Alb, g/dL | 3.7 ± 0.3 | 3.7 ± 0.3 | 0.60 |  | 3.9 ± 0.2 | 3.8 ± 0.3 | 0.12 |
|  | Glb, g/dL | 4.9 ± 0.7 | 4.8 ± 0.7 | 0.66 |  | 4.6 ± 0.8 | 4.9 ± 0.7 | 0.12 |
|  | Crea, mg/dL | 1.1 ± 0.1 | 1.0 ± 0.2 | 0.81 |  | 1.0 ± 0.1 | 1.0 ± 0.2 | 0.56 |
|  | iP, mg/dL | 6.5 ± 1.2 | 6.5 ± 1.2 | 0.72 |  | 6.0 ± 0.9 | 6.3 ± 1.2 | 0.32 |
|  | BCS | 3.3 ± 0.2 | 3.3 ± 0.2 | 0.99 |  | 3.3 ± 0.2 | 3.2 ± 0.2 | 0.45 |

RBC; red blood cell, MCHC; mean corpuscular hemoglobin concentration, TP; total protein, Alb; albumin, Glb; globulin, Crea; creatinine, iP; inorganic phosphate, BCS; body condition score
